# 4000-year-old hair from the Middle Nile highlights unusual ancient DNA degradation pattern and a potential source of early eastern Africa pastoralists

**DOI:** 10.1038/s41598-022-25384-y

**Published:** 2022-12-03

**Authors:** Ke Wang, Madeleine Bleasdale, Charles Le Moyne, Cacilia Freund, Johannes Krause, Nicole Boivin, Stephan Schiffels

**Affiliations:** 1 Max Planck Institute for Evolutionary Anthropology, Leipzig, Germany; 2 School of Life Sciences, Fudan University, Shanghai, China; 3 Department of Archaeology, University of York, York, UK; 4 Max Planck Institute for the Science of Human History, Jena, Germany; 5 School of Social Science, The University of Queensland, Brisbane, Australia

**Keywords:** Genomics, Archaeology, Population genetics

## Abstract

Petrous bones and teeth are the skeletal elements most often targeted by researchers for ancient DNA (aDNA) extraction, and the sources of the majority of previously published ancient African genomes. However, the high temperature environments that characterise much of Africa often lead to poor preservation of skeletal remains. Here, we successfully reconstruct and analyse genome-wide data from the naturally mummified hair of a 4000-year-old individual from Sudan in northeastern Africa, after failed attempts at DNA extraction from teeth, petrous, and cranium of this and other individuals from the Kadruka cemeteries. We find that hair DNA extracted with an established single-stranded library protocol is unusually enriched in ultra-short DNA molecules and exhibits substantial interior molecular damage. The aDNA was nonetheless amenable to genetic analyses, which revealed that the genome is genetically indistinguishable from that of early Neolithic eastern African pastoralists located 2500 kms away. Our findings are consistent with established models for the southward dispersal of Middle Nile Valley pastoral populations to the Rift Valley of eastern Africa, and provide a possible genetic source population for this dispersal. Our study highlights the value of mummified hair as an alternate source of aDNA from regions with poor bone preservation.

## Introduction

The first complete ancient human mitochondrial genome, published in 2008, was generated from the frozen hair of an individual of the Saqqaq archaeological culture from Greenland, dated to 4750 years ago^1^. Since then, the field of ancient DNA (aDNA) research has grown rapidly, with over 6000 ancient genomes published to date. This extraordinary growth has largely been based on analyses of ancient human bones and teeth, with their preservation widely regarded as superior to that of soft tissue and hair^2^. Meanwhile, very few studies have attempted to extract aDNA from hair or soft tissue^1,3^, especially from samples of significant age.

While only accounting for 1.7% of all published genomes, Africa is no exception to this trend. So far, aDNA studies focused on Africa have reported nearly 100 ancient whole genomes, reconstructed from aDNA preserved in petrous bones and teeth from sub-Saharan Africa. Over half of these studies focused on understanding the spread of pastoralism in ancient eastern Africa^4,5^, in particular drawing on samples from present-day Kenya and Tanzania. aDNA studies have revealed multiple waves of human migrations associated with the spread of pastoralism, and complex subsequent admixture events with local hunter-gatherers in eastern Africa^4,5^. Although there is broad consensus that early pastoralists in eastern Africa originated from population dispersals from the north, from regions such as Sudan and Ethiopia^5–7^, no aDNA data related to early pastoralists from these regions has yet been published.

Here, we extract DNA from five specimens (including petrous, teeth and hair) deriving from four individuals from middle Holocene cemeteries in the Dongola Reach of Upper Nubia, northern Sudan. Selected samples for this study represent three individuals from the early 7th millennium BP Neolithic site of Kadruka 21, and one Kerma period individual, directly dated to 4033 cal. BP, from Kadruka 1. Of all the tissues and samples tested, only one sample of preserved hair, from the Kerma period individual, yielded a sufficient amount of authentic ancient human DNA to study. We argue that a critical prerequisite in this extraction was the first application of a state-of-the-art single-stranded library laboratory protocol on hair material, which is able to turn single-stranded DNA molecules into a sequencing library^8^. As we show in our analysis, sequence data generated from hair aDNA using this single-stranded library protocol features unique characteristics that pose challenges in downstream genetic analyses.

Our population genetic analysis of this hair sample from northern Sudan reveals a close genetic affinity of the Kerma period individual to early pastoralists from the Rift Valley in eastern Africa. Drawing on this evidence, as well as previously published proteomic evidence of dietary proteins extracted from the dental calculus of this individual, we conclude that our sample (“Sudan_Kadruka1_4000BP”) represents one of the earliest and most northerly ancient pastoralist populations to have been genetically characterised in Africa to date, as well as potentially a genetic source population for one of the earliest prehistoric pastoralist dispersals into eastern Africa.

## Results

### Archaeological context of Kadruka 1 SK68

Archaeological fieldwork in the Kadruka district of northern Sudan has revealed widespread past occupation of the alluvial plain and paleochannel systems to the east of the current Nile River channel^9^. In accordance with broader archaeological sequences for this region of the Middle Nile Valley^10–12^, cultural deposits were primarily linked to the Neolithic (spanning the 7th millennium BP), as well as the more recent Kerma period (3450–4450 BP). Excavation programs in the Kadruka district focused on several cemeteries from these cultural periods, including Kadruka 1 and Kadruka 21. Reflecting local environmental fluctuations between dry and wet conditions associated with more intensive Nile floods during the Middle Holocene, Neolithic skeletal remains at these cemeteries are typically highly degraded^13,14^. In contrast, progressive local aridification and floodplain contraction^15^, has facilitated enhanced preservation of organics, including hair and leather items, in the more recent Kerma period burials at Kadruka 1^16^.

A recent study exploring the diet of individuals from Kadruka 21 and Kadruka 1 detected milk proteins attributable to domesticated cow (*Bovinae*) or sheep (*Ovis*) in the dental calculus of individual SK129 from Kadruka 21 and to goat (*Carpa*) in the dental calculus of individual SK68 from Kadruka 1^17^. Furthermore, isotopic analyses of hair from individual SK68 (named “Sudan_Kadruka1_4000BP” in this study) broadly indicate a diet primarily composed of C_3_-based resources (C_3_ plants or animals consuming C_3_ plants, δ^13^C -17.0‰, δ^15^N 12.0‰).

The early proliferation of herding economies in northeastern Africa, particularly apparent in the Kerma civilisation of Upper Nubia^11,12^, has been proposed as a potential link to the dispersal of pastoral populations into eastern Africa^5–7^, though there is as yet no published genetic evidence to support this migration model. Direct palaeoproteomic evidence for milk consumption^17^, together with the remains of cattle, sheep and goat found in grave assemblages^18^, identify individuals from Kadruka 1 and Kadruka 21 as belonging to early populations practicing pastoralism, making the sites ideal for archaeogenetic research seeking to examine early pastoralist dispersals in northeastern Africa.

### Ancient DNA authentification and sequencing strategy

**Figure 1:**
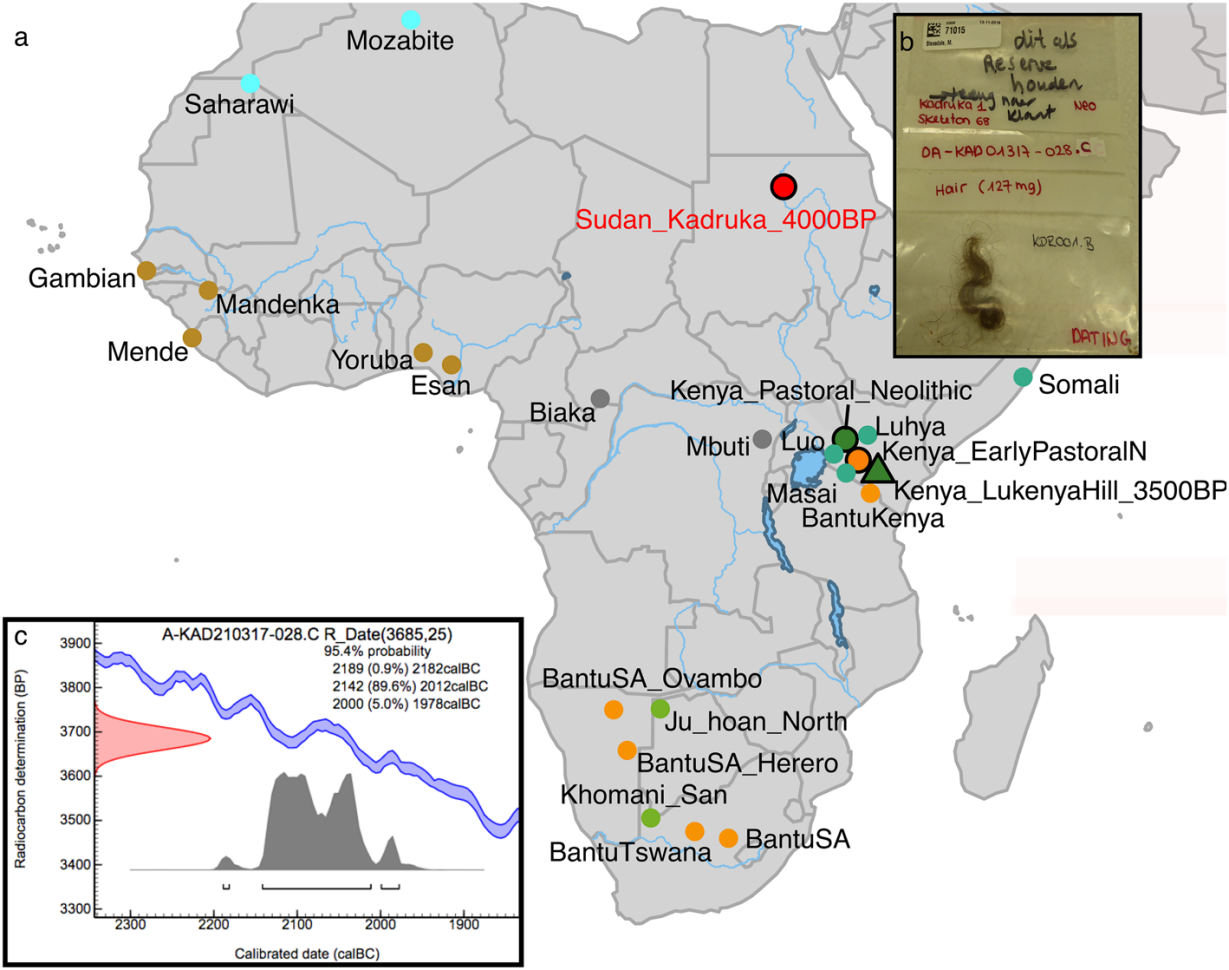
Sample details of the 4,000-year-old hair from Sudan. (**a**) Geographic location of Kadruka and modern African populations used for subsequent analyses. (**b**) Picture of the Kadruka hair sample. (**c**) C14 calibrated age (cal BC) of the Kadruka hair, plotted using IntCal13 calibration curve^19^.

We screened five specimens deriving from four individuals from the Kadruka district of northern Sudan (Fig. 1a) for aDNA preservation, drawing on tooth (KDR001.A), hair (KDR001.B), petrous bone (KDR002, KDR004) and cranial (KDR003) samples. All specimens were excavated from archaeological contexts dating to the Neolithic and Kerma periods. The only sample that yielded detectable authentic aDNA was a lock of dark hair (127 mg) (Fig. 1b) from a Kerma period individual. We used a total of 27.5 cm of hair for aDNA extraction and 78.5 mg for radiocarbon dating. The hair sample from Kadruka 1 SK68 is directly dated to 3928–4139 calBP (Fig. 1c), contemporaneous to the Pastoral Neolithic period in eastern Africa.

To maximize the possibility of aDNA retrieval from shotgun sequencing, we applied double-stranded and single-stranded library protocols to the four extracts deriving from the Kadruka skeletal remains, but only applied the single-stranded library protocol to the hair sample, since this protocol typically results in higher yields than the double-stranded library approach for highly fragmented DNA. None of the aDNA libraries extracted from the skeletal elements showed authentic aDNA damage patterns, i.e., substitutions from cytosine to thymine (C- > T) (Table S1). The hair sample (KDR001.B0101), however, provided an observed 17.5% C- > T substitution rate at the first 5-prime base in sequencing reads (Table S1). Accordingly, we selected this library from the hair sample for deeper sequencing.

We found that the average read length was relatively short (33 base pair/bp, Table S1). This poses a challenge, since short DNA fragments can result in spurious alignments to the human reference genome, even if they do not originate from humans, but from, for example, microbes present in the burial environment. On the other hand, while long DNA fragments have high mapping certainty, they are more likely to originate from modern human DNA contamination (which typically consists of long DNA fragments). We therefore explored various read length cut-offs to yield as much authentic human aDNA as possible, while maintaining a low proportion of reads from presumed modern human contamination. For this purpose, we used two tools to assess both the rate of spurious alignments and the rate of modern human contaminants. First, we used SpAl^20^ which uses simulations to estimate fractions of spurious and authentic alignments given certain read length cut-offs. For a cut-off length of 25 bp, SpAI estimated a spurious alignment fraction of 10% (Table S2). As the read length cut-off increases, the estimation for spurious alignments drops respectively (Table S2). Second, we used AuthentiCT^21^ to estimate the overall contamination level in the aligned fragments using base substitution patterns. We explored length cut-offs at 10, 25, 30 (custom setting) and 34 bp in the raw data processing steps, and summarised EAGER statistics and respective contamination estimates in Table S2.

We find that at a length cut-off of 30 bp, 47.3 ± 2.4% of retrieved aDNA is likely of modern-human contaminant origin (Table 1). In comparison, a length cut-off of 25 bp yielded 4,680,356 mapped reads with 0.1 ± 0.3% contamination (Table 1). Together with the results from SpAL, we consider 25 bp a safe cut-off length for this library. Thus, we continued our downstream analyses with a 25 bp read length filter, ending up with 231,040 sequencing reads after mapping, from which we derived 3,336 pseudo-haploid allele calls on 1240 k SNP positions (Table 1).

**Table 1:** Eager statistics of deeper shotgun sequenced data from the hair sample KDR001.B0101.SG1.2 with different length filter cut-off.

Read length filter	Nr. raw reads	Nr. reads prior mapping	Nr. Mapped reads (no qc)	Nr. Mapped reads(q30)	Nr. on-target (1240 k) reads	Nr. called 1240 K SNPs	Nr. MT reads	Mt/NUC ratio	Nr. reads on X:Y (q30)	Damage 1st base 3’ %	Damage 2nd base 3’ %	Damage 1st base 5’ %	Damage 2nd base 5’ %	Median fragment length	AuthentiCT contam %
30 (default)	99,830,433	2,606,646	97,432	52,949	1113	952	172	329.78	1632:84	0.74	0.43	16.23	11.64	32	47.3 ± 2.4
25 (preferred)	99,830,433	4,680,356	576,384	231,040	5464	3336	656	224	6581:405	1.06	1.03	15.9	11.76	26	0.1 ± 0.3

We performed contamination tests using AuthentiCT, which is designed for sequences from single-stranded libraries.

### Characteristics of the aDNA fragments from hair

Employing our final read length filter at 25 bp and additionally filtering for alignment mapping quality (Methods), we further explored alignment statistics. We find two unusual characteristics in the aDNA library generated from the Kadruka hair sample. The first is that the sample is enriched in unusually short DNA molecules, giving a median read length of 25 bp, compared to 44 bp for typical bone-derived shotgun aDNA in a previous African aDNA study using the same laboratory pipeline^4^. The second is that unusually high damage rates were observed in the interior of the DNA molecules from hair, while unusually low damage rates were seen in the exterior of the molecules. For instance, at the interior 10th bp from the 5’ end, damage rates were 10% compared to 1% from typical bone-derived aDNA. While at the exterior, the 1st bp from the 5’ end showed damage rates of 15%, compared to on average of 27%^22^ from typical bone-derived aDNA. These patterns are consistent with high degradation of DNA fragments in hair through intense sun exposure, already during the lifetime of the individual, which may result in hair containing largely denatured single-stranded DNA fragments, as opposed to the more typically intact double-stranded fragments preserved in bone samples.

**Figure 2:**
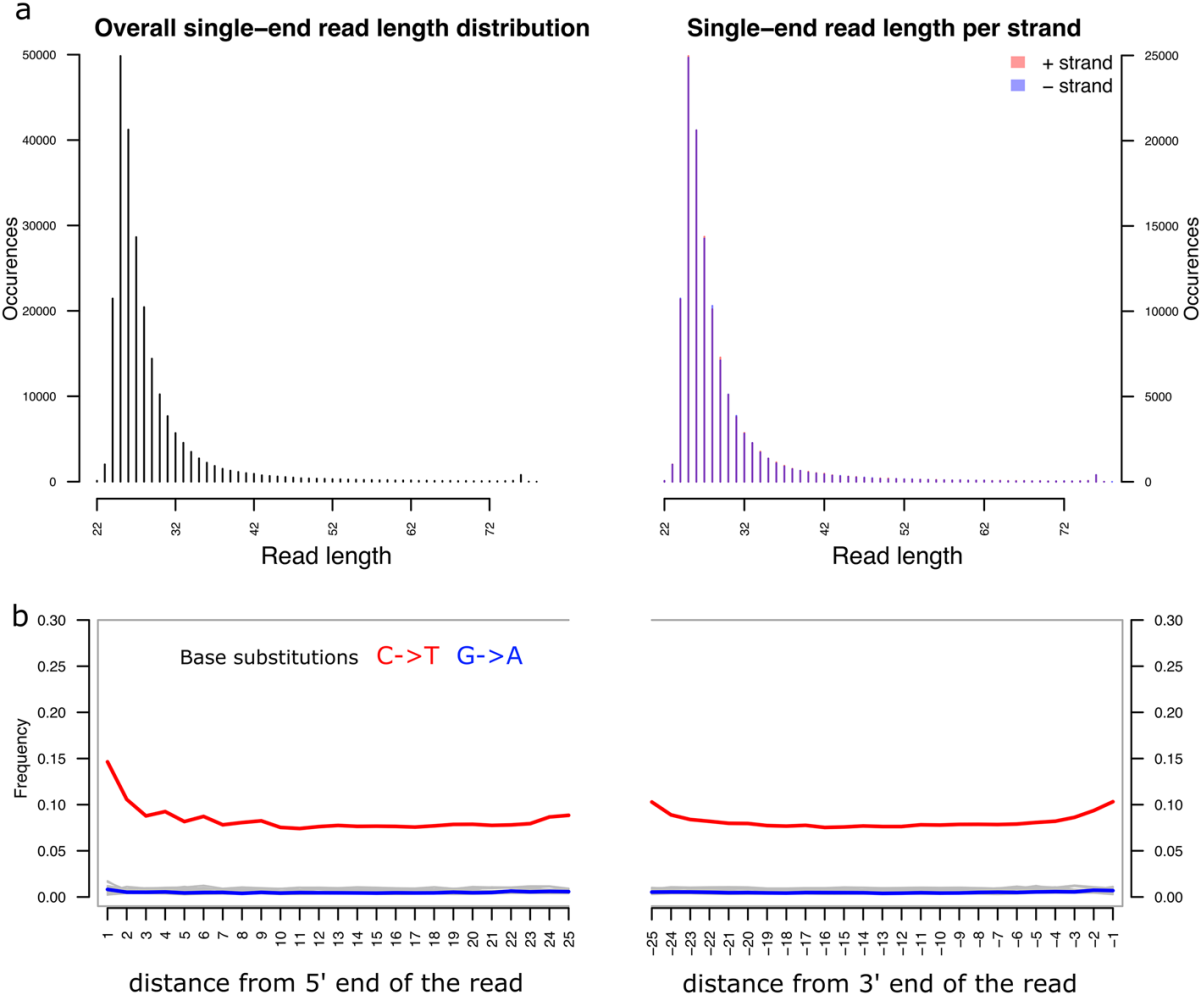
Characteristics of aDNA fragments from the hair sample. (**a**) Length distribution of shotgun sequencing reads mapped to the whole genome using read length filter cut-off at 25 bp in the step of adaptor removal. (**b**) High C-to-T substitution rates in the interior of aDNA fragments.

We find the ratio of mitochondrial to nuclear DNA in the hair-derived DNA library to be relatively high (with a ratio at 224 at length cut-off 25 bp, Table S1), compared to typical rates with other tissues; for example the ratio is at 110 on average for petrous bones in a previous study using the same laboratory pipeline^4^. We investigated whether there is a notable difference in terms of aDNA preservation in nuclear DNA and mitochondria from the hair material. Specifically, we examined if the two idiosyncratic features of hair aDNA we describe above apply to both nuclear and mitochondrial DNA from hair. In Fig. 2 and Fig S2, we compared the read length distribution and average base substitution rates of reads mapped to the complete genome (i.e., nuclear and mitochondrial), the nuclear genome, and the mitochondrial genome. We find that both nuclear and mitochondrial DNA have high base substitution rates in the interior of sequence reads (Fig S2a, c), but reads mapped to mitochondria are relatively longer than reads mapped to autosomes (Fig S2b, d).

Given the tenfold reduction of mapped reads after applying a mapping quality filter (Table 1), we examined if the two features of hair aDNA observed here resulted from the mapping quality filter. We find that our mapping quality filter did not have a notable effect on the two features of ultra-short DNA fragment enrichment and high interior aDNA damage pattern (Fig S3).

Given the success of SNP capture techniques for poorly preserved human DNA^23^, we also performed SNP capture for our hair-derived aDNA library. However, SNP capture did not provide an improvement over shotgun sequencing. Instead, we found the base substitution rates in the exterior and interior of reads are substantially lower in capture data, in comparison to the rate distribution in shotgun data (Fig. S1, Table S1), corroborating the fact of high contamination rate in the capture data (42 ± 3% as estimated by *AuthentiCT*), likely due to capture preferentially targeting molecules without damage (due to more effective hybridization) than with damage. In addition, longer molecules are preferentially captured over short molecules.

### Genetic affinity to early eastern African pastoralists

**Figure 3:**
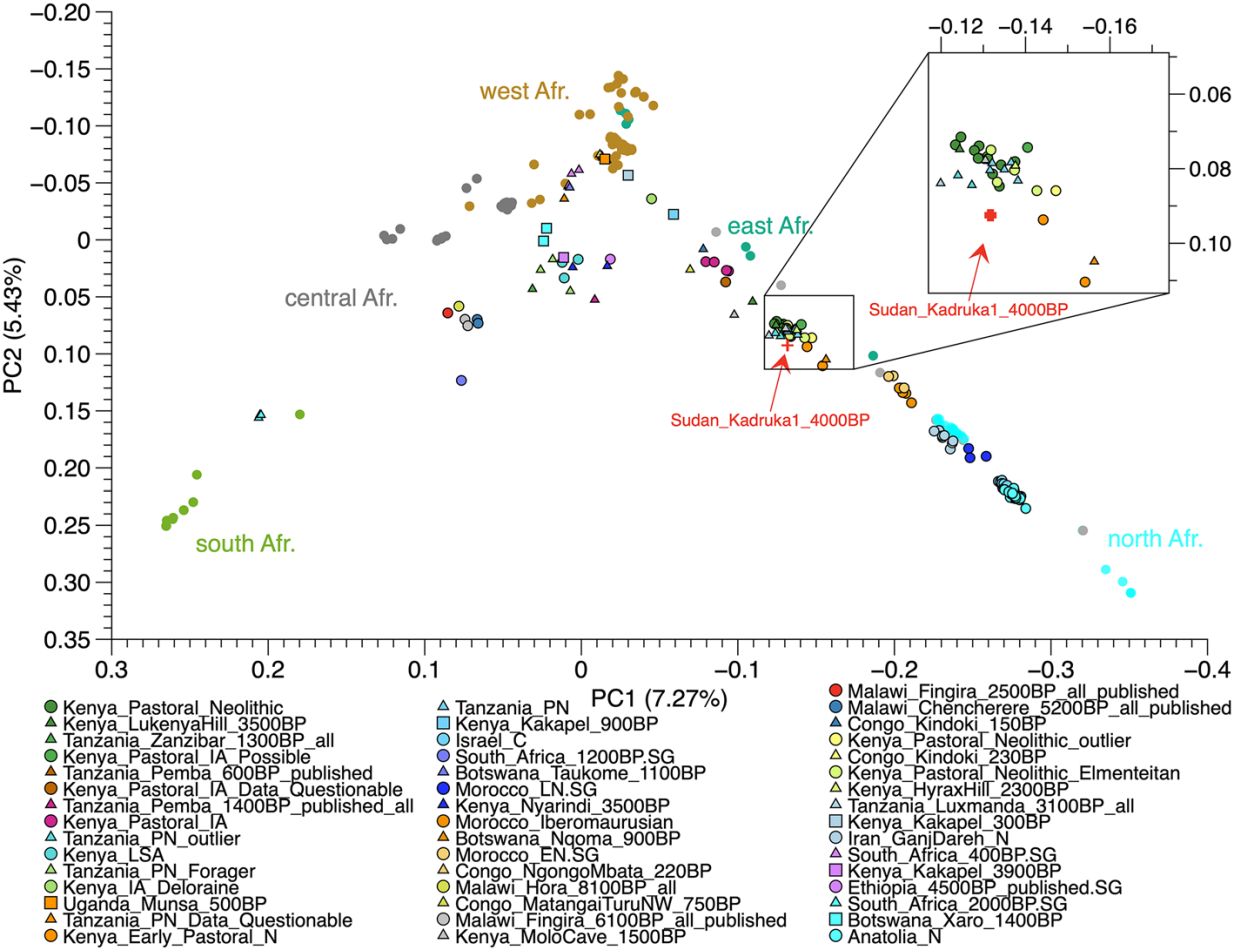
Genetic ancestry of Sudan_Kadruka1_4000BP. Principal Component Analysis (PCA) of African populations. We project Sudan_Kadruka1_4000BP into an African PCA (Table S3) with PCs calculated from modern Africans in SGDP^24^. We use block jackknife strategy (taking-one-chromosome-out)^26^ for error bar calculation of Sudan_Kadruka1_4000BP ‘s location on the PCA.

We performed Principal Components Analysis (PCA) and Outgroup-*f3* (Figs. 2, 3) to investigate the genetic ancestry of the individual (Sudan_Kadruka1_4000BP) from whom our hair sample derived, utilizing 3336 mapped reads overlapping with SNP positions from the Shotgun data, after read length filter at 25 bp of Sudan_Kadruka1_4000BP (Table S2). To maximise the resolution given the extremely low coverage and low number of called alleles, we used high-coverage modern African genomic data from the SGDP^24^ and the HGDP^25^, which includes all SNPs in 1240 k panel, instead of the commonly used Human Origin array data^26^, for calculating Principal Components (PCs). We projected ancient Africans and ancient Near Easterners on the background of modern African groups^25^. Although the number of available populations in SGDP and HGDP is limited, we observe clear separations of African populations from different regions, with eastern/northern, southern and western African populations falling into the right, left and top corner of PC1/PC2 space, respectively.

The PCA shows Sudan_Kadruka1_4000BP located close to previously published early pastoralists in eastern Africa^4,5^, such as Kenya_EarlyPastoral_N (3800–4000 calBP) and Kenya_Pastoral_Neolithic (1500–3000 calBP). Kenya_EarlyPastoral_N is a group of two pastoralist individuals dated to the early stage of the eastern African Pastoral Neolithic, both of whom are genetically derived from admixture between two early northeastern African-related ancestries from Sudan and Northern Africa/Levant^5^. To estimate the level of noise resulting from the sparsity of our SNP data, we computed a standard error for the projected PCs of Sudan_Kadruka1_4000BP using a block jackknife approach. Specifically, we computed pseudo-values by deleting each chromosome of the genotype data in turn and then used the resulting estimates from the remaining data as input for the weighted jackknife calculation^27^. We find the standard errors of Sudan_Kadruka1_4000BP to be relatively small compared to overall genetic variation within Africa, which gives us confidence that the location calculated from the full data (Fig. 2a) is robust.

**Figure 4:**
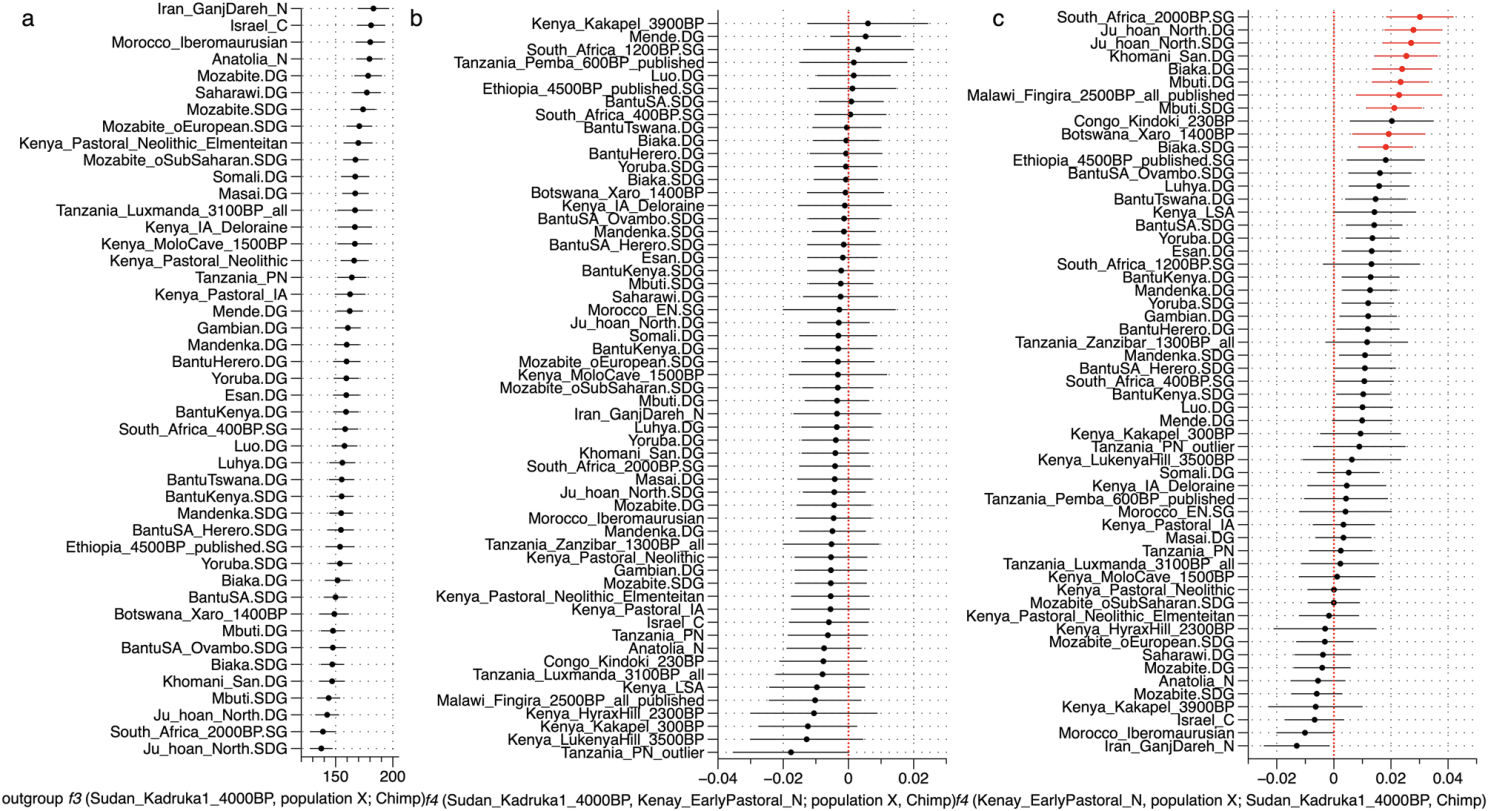
Genetic affinity with ancient African pastoralists. (**a**) *Outgroup f3*(Sudan_Kadruka1_4000BP, population X; Chimp). (**b**) *f4*(Sudan_Kadruka1_4000BP, Kenya_EarlyPastoral_N; population X, Chimp). (**c**) *f4*(Kenya_EarlyPastoral_N, population X; Sudan_Kadruka1_4000BP, Chimp). Population X includes published ancient African and Near Eastern populations and modern African populations from SGDP and HGDP data sets (Table S3). We plot two standard error bars for *f3* and *f4* statistics shown here and highlight statistically significant tests (Z-score > 3) in red color.

Despite the sparsity of the data, the PCA analyses conducted here clearly suggest a very close genetic relationship between Sudan_Kadruka1_4000BP and ancient eastern African pastoralist populations. To corroborate this finding, we also computed allele sharing rates with ancient populations from the Levant and Africa and present-day African populations at genomic sites where Sudan_Kadruka1_4000BP differs from the chimpanzee reference genome via outgroup-*f3* (Sudan_Kadruka1_4000BP, population X; Chimpanzee). Figure 4a shows that Sudan_Kadruka1_4000BP shares the highest genetic affinity with ancient Levantine groups, ancient northern and Eastern Africans and modern Africans from northern Sahara and the Horn of Africa. We computed pairwise comparisons employing *f4* (Sudan_Kadruka1_4000BP, Kenya_EarlyPastoral_N; population X, Chimp) to validate the close PC location between Sudan_Kadruka1_4000BP and Kenya_EarlyNeolithic_N. Consistent with PCA location, *f4*-statistic result confirms the genetic cladality between Sudan_Kadruka1_4000BP and Kenya_EarlyPastoral_N given that none of the tested populations breaks cladality significantly, suggesting that these two individuals are indistinguishable in terms of allele frequencies (Fig. 4b). Additionally, we show that in f4 (Kenya_EarlyPastoral_N, population X; Sudan_Kadruka1_4000BP, Chimp) results (Fig. 4c) that all tested ancient and modern African populations are either significantly positive (suggesting that they are less close to Kenya_EarlyPastoral_N compared to Sudan_Kadruka1_4000BP) or overlapping with zero, indicating equal genetic distance to both.

## Discussion

In this study, we demonstrate the possibility of retrieving aDNA from ancient hair by reconstructing genome-wide data from a 4000-year-old hair sample in Sudan. We find that aDNA extracted from hair preserved in this extremely hot and arid environment features unusually short DNA fragments and unusually high interior aDNA damage patterns. We interpret such characteristics as a combined effect of the unique preservation environment in Sudan, of hair as a unique aDNA source, and of the single-stranded library protocol that we applied to generate our sequencing library. The high regional temperature likely leads to i) denaturation of double-stranded DNA molecules to single-stranded DNA, and ii) more frequent breakage of DNA molecules, resulting in shorter DNA fragments. Hence the predominance of short, single-stranded DNA in hair explains the excess C- > T substitution rate in the interior of sequencing reads. Meanwhile, the arid preservation environment and the specific properties of hair potentially reduce enzymatic activity, resulting in reduced hydrolysis damage^28^, preventing the commonly-observed high degradation at the 5’ end of aDNA fragments and explaining the relatively low substitution rate at the 5’ end of the ancient hair DNA molecules.

Given these properties, the single-stranded library protocol we employed is uniquely well-suited as a sequencing tool in this case, maximising library complexity from heavily degraded and in large parts single-stranded source DNA fragments by building library molecules from both forward and backward strand DNA fragments regardless of the DNA strand orientation. In such cases, both short and long DNA fragments are sequenced effectively. Hence, our shotgun sequencing on the single-stranded library accumulates the short DNA fragments, as reflected in the read length distribution with a peak around short read lengths, and keeps the long fragments with interior damage, as reflected in the average high interior damage with C->T substitution rate over 10% throughout the sequence read (Fig. 2).

To our surprise, only the DNA from hair, rather than from the tooth of the same individual, showed signatures of authentic human aDNA preservation, as seen from the pattern of C- > T substitutions, regardless of rather similar endogenous DNA content in tooth and hair (Table S1). From the hair sample, we obtained 231,040 mapped reads after applying mapping and base quality filters on nearly 100 million raw reads (Table 1), corresponding to 0.231% endogenous DNA from shotgun whole genome sequencing. One consequence of the relatively short DNA fragments in sequencing libraries built from hair is that the commonly used 1240 k SNP capture technique failed to enrich authentic human aDNA. Instead, in our case, 1240 k capture likely enriches for long non-deaminated sequences which are non-authentic aDNA (Fig S2). Consequently, we generated and analysed whole genome shotgun data throughout this study, which is less cost-effective than capture technology.

The very low coverage data resulting from this unique sample posed a challenge for downstream population genetic analyses. Nevertheless, we see from *f3-statistics* that this individual, from a rural agro-pastoral population linked with the Kerma culture of Upper Nubia^16^, shares close genetic affinity with Levantine groups. Moreover, we could show that this individual is genetically indistinguishable from early Pastoral Neolithic individuals dated to 4000BP living over 2500 km away in Kenya and Tanzania, even when correcting for relatively large standard errors in population genetic estimates due to the low coverage. This close relatedness to early pastoral populations in eastern Africa is consistent with archaeological evidence for the dispersal of herding populations southwards along the Nile River Valley following their establishment in the Kadruka region from the early 7th millennium BP^29,30^, although we caution that inference from a single sample can at best be tentative.

The high affinity of the Kerma period individual from Kadruka 1 with Neolithic pastoral groups far to the south, and genetic indistinguishability of their sequenced DNA, would be consistent with this sample representing a possible genetic source population for the earliest eastern African pastoralists who settled in the Rift Valley. This in turn would point to a high degree of mobility of pastoralists between the Middle Nile Valley and present-day Kenya, potentially before or around the Pastoral Neolithic. It would imply that the southward dispersal of pastoralists from the Middle Nile Valley did not involve genetic exchange with pre-existing human groups along the migration route, particularly local foragers, and may therefore have been relatively rapid. Indeed, archaeological research suggests that dispersals were likely driven in part by increasing population pressure and regional aridification, with a marked period of aridity in Upper Nubia preceding the first appearance of pastoral groups in the Turkana Basin of eastern Africa at 5000 BP^29–31^. These early eastern African pastoralists at sites like Lothagam in the Turkana Basin^29,32^, while featuring regional forms of cultural expression, also broadly maintained traditions of monumental mortuary expression, as well as traditions of burial with elaborate grave goods for which the Neolithic and Kerma period cemeteries of the Middle Nile Valley, including Kadruka 1, are recognised^9^.

While much archaeogenetic research in Africa has focused on the Neolithic populations of eastern Africa, their source populations have, from a genetic standpoint, remained elusive. Highlighting the need for additional aDNA from ancient northeastern African populations, the aDNA recovered from ancient human hair in this study provides important new genetic data relevant to the detailed reconstruction of early pastoral dispersals from northeastern to eastern Africa. Our findings also point to the value of preserved ancient hair as a source of aDNA, particularly in regions where more typical aDNA archives are unavailable or poorly preserved, with exciting implications for future aDNA research.

## Methods

### aDNA extraction and C14 dating on the mummified hair

We sequenced five specimens (four individuals) in total from Kadruka 21 and Kadruka 1 for aDNA in dedicated clean rooms at the Max Planck Institute for the Science of Human History in Jena, Germany. We constructed both single-stranded and double-stranded DNA libraries for skeleton remains and single-stranded library for the hair sample following previously published protocols^8,33^, and performed both whole genome shotgun and 1240 k-hybridization capture^23^ to maximise the amount of aDNA.

We sampled from a mixed composition of hair for aDNA analyses and report the direct C14 date for the hair sample from separate additional sampling. A bulk hair sample was sampled for dating at the Centre for Isotope Research (CIO) Groningen in 2019. A sample of 78.5 mg of hair was chemically treated with 4% HCl, rinsed with decarbonized H_2_O and dried before combustion. Combustion and graphitization of a subsample of the pre-treated hair (5.35 mg) was carried out according to the methods outlined in Dee et al. (2020)^34^. The ^14^C measurements were carried out using an Ionplus AMS-MICADAS^35,36^. C14 ages are calibrated to calendar years with OxCal version 4.3^37^, using calibration curve IntCal13^19^.

### Bioinformatic processing

We processed DNA sequences using the EAGER v1.92.56^38^, with adaptors removed by AdapterRemoval v2^39^ using flags *AdapterRemoval –threads 4 –trimns –trimqualities –preserve5p –minlength 25 –minquality 20 –minadapteroverlap 1*, reads mapped to hs37d5 by BWA v0.7.12^40^ using flags *bwa aln -t 4 -n 0.01 -l 32* , and polymerase chain reaction duplicates removed by Dedup software v0.12.2^38^. mapDamage v2.0.9^41^ was used to calculate substitution rate in read termini of sequenced DNA fragments.

We estimated the amounts of spurious alignments at various read length cut-off using SpAI (bioinf.eva.mpg.de/SpAl/). We tested three different read length filters (34, 25, 10 bp) to yield the maximum efficiency for endogenous aDNA while assuring contamination free. We used AuthentiCT^21^, which is designed for single-stranded library DNA sequences, to examine the contamination level. Finally, we applied a minimum read length filter of 25, and a minimum base quality of 30 and a minimum mapping quality of 30 for genotype calls. We call pseudo diploid genotypes by randomly sampling an allele for each site in the 1240 k panel using *Samtools v1.3*^42^ and pileupCaller (github.com/stschiff/sequenceTools).

### Population genetics analyses

For downstream population genetic analyses, we combined our new data with previously published ancient African and Near Eastern genomes^4,5,22,43–50^, together with 30 high-coverage modern African populations from the Simons Genome Diversity Project (SGDP)^24^ and Human Genome Diversity Project (HGDP) datasets^25^.

We performed PCA using smartpca v16000^51^ with “numthreads: 8” and “lsqproject: YES” option in the parameter file, and projected ancient individuals into the PC space calculated from modern African populations in SGDP (Table S3)^24^. No shrinkmode option was applied, and the default outlier removal with up to 5 iterations was not altered. We applied a block jackknife approach for calculating standard errors (SE) of the PC location of Sudan_Kadruka1_4000BP^52^. We dropped one chromosome (i.e. block) each time and iterated for 22 chromosomes, to get an estimate of SE.

We calculated *f3* and *f4* statistics using qp3pop v435 from the AdmixTools v5.1 package^26^ using options “*outgroupmode: YES*”, and qpDstat v755 from the same package^26^ using options “*f4mode: YES*” and “*printsd: YES*”. We performed the Outgroup *f3* (Sudan_Kadruka1_4000BP, population X; Chimpanzee) test using Chimpanzee as the outgroup population. Population X represents published ancient African and Near Eastern populations and high-coverage modern populations from SGDP and HGDP datasets documented in Table S3. We report *f3* test results using more than 1000 overlapping SNPs. Given that Sudan_Kadruka1_4000BP shares high genetic affinity with early eastern African pastoral groups, we further tested the same list of population X using *f4* (Sudan_Kadruka1_4000BP, Kenya_EarlyPastoral_N; population X, Chimp) and *f4* (Kenya_EarlyPastoral_N, population X; Sudan_Kadruka1_4000BP, Chimp) for the genetic cladility between Sudan_Kadruka1_4000BP and Kenya_EarlyPastoral_N.

## Supplementary Information

Supplementary Information 1.

Supplementary Information 2.

## Data Availability

Genomic data newly generated in this study (BAM and original fastq file) are available through the European Nucleotide Archive (ENA), with accession number: PRJEB53198.
