# Supplementary material for: 4000-year-old hair from the Middle Nile highlights unusual ancient DNA degradation pattern and a potential source of early eastern Africa pastoralists
Source: Sci Rep. 2022 Dec 3;12:20939. doi: 10.1038/s41598-022-25384-y (PMC9719486; doi:10.1038/s41598-022-25384-y)
Supplement: Supplementary file 1 — Supplementary Information 1. [file 41598_2022_25384_MOESM1_ESM.docx]

**Supplementary Figures and Tables**

**Table S1**: Sequencing details of five screened specimens

**Table S2**: Investigation of the proper read length filter cut-off with shotgun screening sequence data of Sudan_Kadruka1_4000BP

**Table S3**: List of ancient genetic groups used for analyses and related publications

**Fig S1**: Comparison of aDNA damage pattern from shotgun and capture data

**Fig S2**: Comparison of read length distribution, damage pattern of reads mapped to autosomes only, and mitochondria only

**Fig S3**: Comparison of read length distribution, damage pattern of reads mapped to whole genome with and without mapping quality filter

| Sample ID (Archaeology ID) | Library type | Nr. Raw Reads | Nr. Mapped Reads | Nr. MT Reads | Mt/NUC Ratio | Damage 1^st^ Base 5’ | Damage 2^nd^ Base 5’ | Median fragment length |
| --- | --- | --- | --- | --- | --- | --- | --- | --- |
| KDR001.A (Kadruka 1/SK68) | ds | 5,090,659 | 10,971 | 3 | 55.4 | 1% | 0.30% | 64 |
|  | ss | 6,173,153 | 9,525 | 2 | 57.4 | 2.70% | 1.30% | 40 |
| KDR001.B (Kadruka 1/SK68) | ss | 5,600,328 | 8,095 | 10 | 278.8 | 17.50% | 12.20% | 33 |
|  | ss + 1240k capture | 17,656,718 | 190,672 | 179 | 200.240 | 0.2% | 3.3% | 37 |
| KDR002 (Kadruka 21/SK129) | ds | 4,106,569 | 1,360 | 0 | 0 | 2.10% | 2% | 31 |
|  | ss | 8,581,866 | 2,144 | 0 | 0 | 3% | 1.30% | 31 |
| KDR003 (Kadruka 21/ SK129) | ds | 24,660,449 | 18,118 | 5 | 59.2 | 1.10% | 0.30% | 71 |
|  | ss | 5,630,029 | 8,703 | 3 | 61 | 1.60% | 0.60% | 52 |
| KDR004 (Kadruka 21/SK10) | ds | 4,246,906 | 10,639 | 9 | 152 | 0.80% | 0.30% | 60 |
|  | ss | 6,734,623 | 12,949 | 12 | 217.4 | 1% | 0.80% | 45 |

**Table S1**. Sequencing details of five screened specimens (shotgun screening sequenced approximately 5 million reads for each). Each sample (except the hair) has been sequenced twice, using both double-stranded (ds) and single-stranded (ss) library protocols. We built ss library for the hair sample and tried both whole genome shotgun sequencing and 1240k capture sequencing. We apply the default read length filter 30bp during adaptor removal to get the summary statistics shown in the table.

(a). Estimated fraction of the spurious alignments conditional

| length cut-off (bp) | Spurious Alignment Fraction (%) | 95% Confidence Interval |
| --- | --- | --- |
| 20 | 1 | (0.9441,1) |
| 21 | 0.9688 | (0.9369,0.9873) |
| 22 | 0.9515 | (0.9127,0.9764) |
| 23 | 0.1522 | (0.1109,0.2016) |
| 24 | 0.32 | (0.2169,0.4378) |
| 25 | 0.1053 | (0.0502,0.1884) |
| 26 | 0.1324 | (0.0561,0.251) |
| 27 | 0.0507 | (0.0066,0.168) |
| 28 | 0.0241 | (0.0007,0.1243) |
| 29 | 0.0829 | (0.0108,0.2641) |
| 30 | 0.0517 | (0.0015,0.2512) |
| 31 | 0 | (0,0.2646) |
| 32 | 0 | (0,0.2646) |
| 33 | 0 | (0,0.4096) |
| 34 | 0.2576 | (0.0072,0.8107) |
| 35 | 0 | (0,0.6024) |

(b.) Summary statistics of the aDNA damage and contamination information

| Read length filter cutoff | Nr. Raw reads | Nr. Mapped Reads | Damage 1^st^ Base 5’ % | Damage 2^nd^ Base 5’% | AuthentiCT Contamination% | AuthentiCT St. Err.% |
| --- | --- | --- | --- | --- | --- | --- |
| 10 | 5,600,328 | 2,782,738 | 1.3 | 1.13 | 22.0 | 1.93 |
| 25 | 5,600,328 | 36,635 | 16.06 | 12.29 | 0.1 | NaN |
| 30 (default) | 5,600,328 | 6,335 | 16.08 | 11.08 | 36 | 1.7 |
| 34 | 5,600,328 | 2,769 | 9.41 | 6.46 | 37.5 | NaN |

**Table S2**. Investigation on the proper read length filter cut-off for the shotgun sequencing data from hair DNA. (a). Estimated fraction of the spurious alignments conditional on various read length cut-off. We summarise the column of “SpuriousAln_SF (95%CI)” from the SpAI estimates, which shows the fraction of spurious alignments at the corresponding length cut-off with 95% confidence intervals shown as error bars at each bin. (b). Summary statistics of the aDNA damage and contamination information when applying read length cutoff at 10, 25, 30, 34 base pair during adaptor removal.

**Table S3**. See separate excel table.


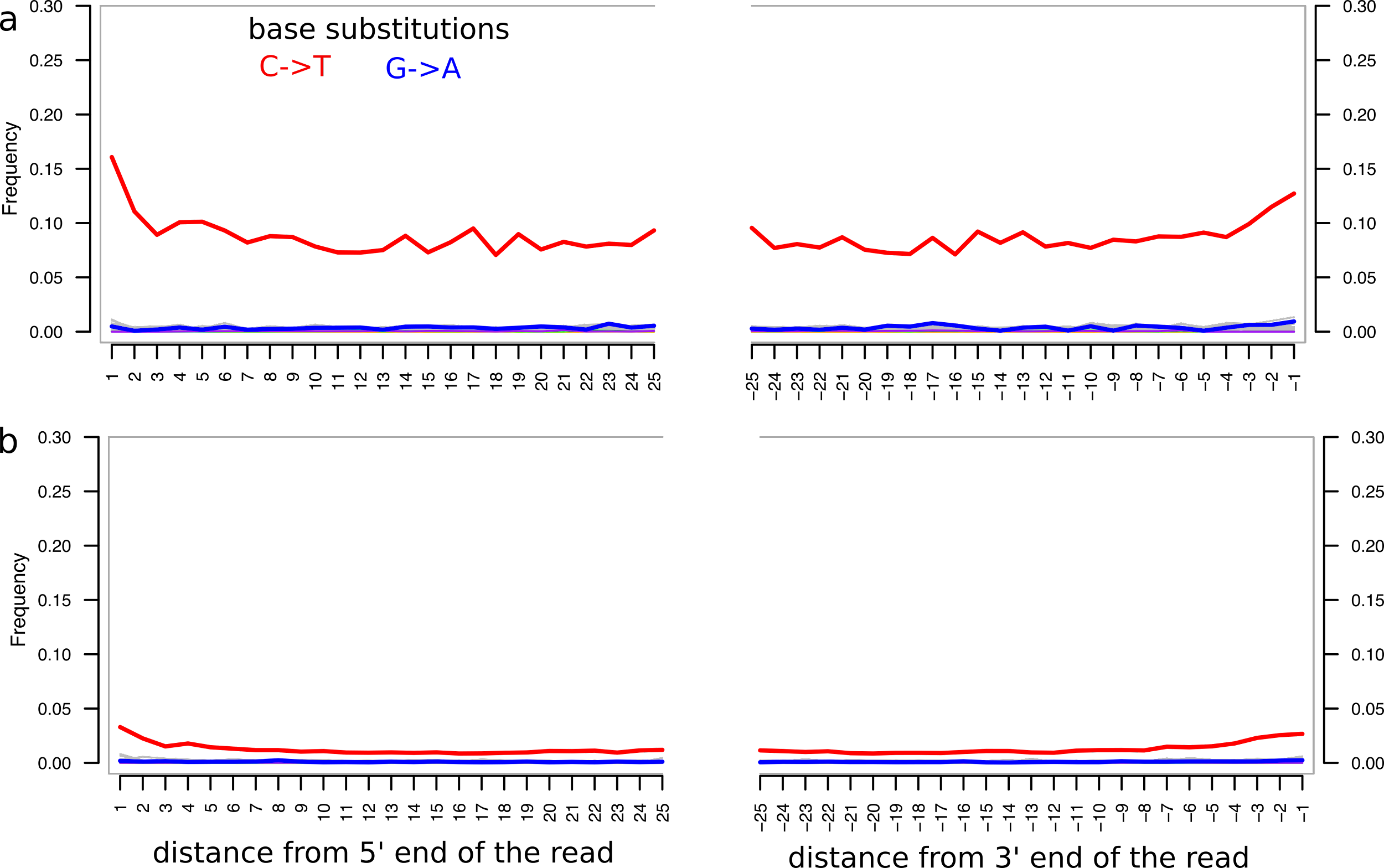


**Figure S1.** Comparison of aDNA damage pattern from shotgun and capture data. **a**. damage pattern of shotgun sequencing data. **b**. damage pattern of capture sequencing data.


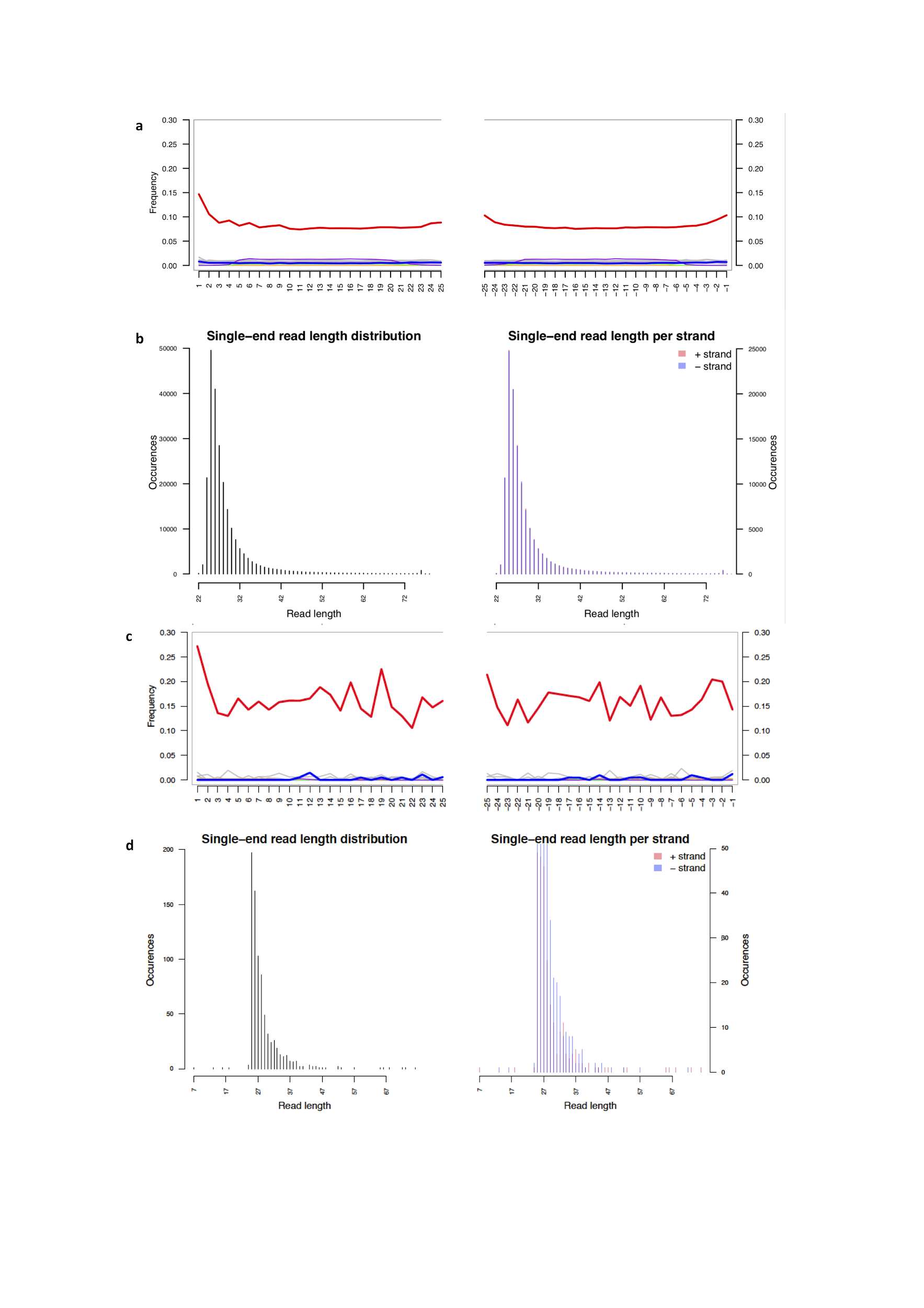


**Figure S2**. Comparison of read length distribution, damage pattern of reads mapped to autosomes only, and mitochondria only. **a**. Damage pattern based on reads mapped to nuclear only (q30, 229 302 reads) **b.** Read length distribution based on reads mapped to nuclear only (q30, 229 302 reads). **c.** Damage pattern based on reads mapped to mitochondria only (q30, 785 reads). **d.** Read length distribution based on reads mapped to mitochondrial only (q30, 785 reads)


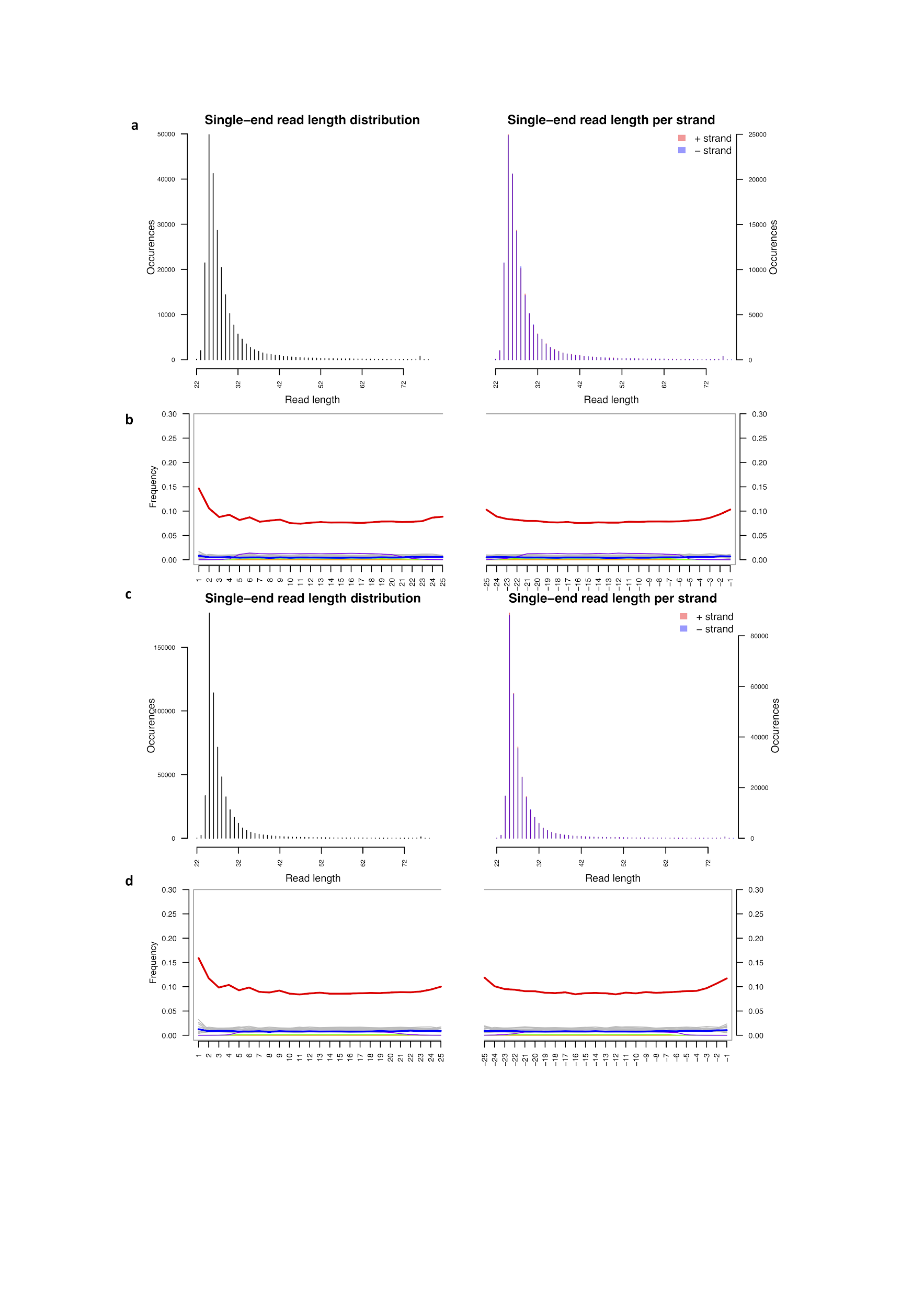


**Figure S3**: Comparison of read length distribution, damage pattern of reads mapped to whole genome with and without mapping quality filter. **a.** Damage pattern based on reads mapped to the whole genome after mapping quality filter (q30). **b.** Read length distribution based on reads mapped to the whole genome after mapping quality filter (q30). **c**. Damage pattern based on reads mapped to the whole genome without any mapping quality filter. **d.** Read length distribution based on reads mapped to the whole genome without any mapping quality filter.
